# Synthesis, Characterization, and Pesticidal Activity of Emamectin Benzoate Nanoformulations against *Phenacoccus solenopsis* Tinsley (Hemiptera: Pseudococcidae)

**DOI:** 10.3390/molecules24152801

**Published:** 2019-08-01

**Authors:** Asem Elabasy, Ali Shoaib, Muhammad Waqas, Mingxing Jiang, Zuhua Shi

**Affiliations:** 1 Key Laboratory of Molecular Biology of Crop Pathogens and Insects, Ministry of Agriculture, Institute of Insect Sciences, College of Agriculture and Biotechnology, Zhejiang University, 866 Yuhangtang Road, Hangzhou 310058, China; 2 Department of Pesticides, Plant Protection Research Institute, Agricultural Research Center, Cairo 11341, Egypt

**Keywords:** emamectin benzoate, cellulose nanocrystals, silicon dioxide nanoparticles, delivery system, *Phenacoccus solenopsis*, biological activity

## Abstract

Using nanotechnology to develop new formulations of pesticides is considered a possible option in enhancing the efficiency, safety, and photostability of pesticides under various climatic conditions. In the present study, two novel nanoformulations (NFs) were successfully prepared based on nano-delivery systems for emamectin benzoate (EMB) by loading it on cellulose nanocrystals (CNCs) and silicon dioxide nanoparticles (SNPs) as carriers through a freeze-drying method. The synthesized nanoformulations were examined using field emission scanning electron microscopy (FE-SEM), transmission electron microscopy (TEM), X-ray diffraction (XRD), Fourier transform infrared spectroscopy (FTIR), thermogravimetric analysis (TGA), and dynamic light scattering (DLS). The results showed that SNPs and CNCs had a loading efficiency of 43.31% and 15.04% (*w*/*w*) for EMB, respectively, and could effectively protect EMB from photolysis under UV radiation. The LC_50_ values for EMB + SNPs, EMB + CNCs, and EMB commercial formulation against *Phenacoccus solenopsis* were 0.01, 0.05, and 0.31 μg/mL, respectively, indicating that both NFs were more effective than the EMB commercial formulation. This work seeks to develop new nano-carriers for potential applications of pesticides in plant protection, which will reduce the recommended dose of pesticides and thereby decrease the amount of pesticide residue in food and the environment.

## 1. Introduction

Pesticides play a crucial role in agriculture to obtain high crop productivity and adequate food supplies. However, repeated and indiscriminate application of pesticides has led to numerous issues, including increasing health risks to humans and animals, killing nontarget animals (e.g., predators and parasitoids of insect pests, and pollinators), polluting the environment, and increasing pest resistance [1]. Conventional pesticide formulations have several drawbacks, including poor solubility in water, high sensitivity to photolysis/hydrolysis, and easiness of evaporating and drifting along with the wind [2,3]. As a result, more than 90% of pesticide formulations run off into the environment instead of reaching target sites, which reduces the pesticide-use efficiency and meanwhile worsens the pesticide-related issues [4]. Therefore, it is essential to develop new routes and nanomaterials to improve pesticide formulations and to overcome their drawbacks.

Recently, the advancements of nanotechnology and nanomaterials have given new ways of improving the efficiency and safety of pesticides through constructing a nano-delivery system and using nanomaterials as carriers [5,6]. Pesticide nanoformulation (NF) delivery systems possess a lot of remarkable properties, such as high solubility, wettability, adhesion, and penetration to the surface of target insects, due to their small size and high surface area [7,8]. Cellulose is one of natural polysaccharides and biopolymers widely used in the preparation of novel compounds [9]. Nanocellulose, a promising renewable material, has gained much interest in recent years. Currently, it has been used in many fields, i.e., food industry, pharmaceutical industry, cosmetic additives, packaging, and hygiene products, etc. [10] because of its low cost, large surface area, thermal stability, excellent colloidal stability, and biodegradability. Also, nano-cellulose has emerged as a promising adsorbent with many applications in environmental studies and wastewater treatment because it has high adsorption potential for organic and inorganic materials [11]. Cellulose nanocrystals (CNCs) have a low eco-toxicological hazard and are accepted as safe nanomaterial by Environment Canada’s Domestic Substance List [12,13]. However, there is no report about the use of CNCs as carrier materials in the field of pesticide.

Silica-based nanomaterials have been gaining more attention in recent years as a potential delivery agent for biomedical and agricultural applications. This is mainly because of their structural flexibility in forming nanomaterials of different sizes and shapes, low cost, large surface area, and non-toxicity and has also been approved by the U.S. Food and Drug Administration as a safe material [14,15,16]. Moreover, Silicon dioxide nanoparticles (SNPs) have been demonstrated to be capable of improving the dispersity, bioavailability, and efficiency of pesticide compounds and in overcoming the physiological resistance of pests [17,18].

Emamectin benzoate (EMB) is a 4’-deoxy-4’-methylamino derivative of abamectin, a 16-membered macrocyclic lactone produced via the fermentation of the soil microorganisms, *Streptomyces avermitilis*. It consists of a mixture of approximately 90% 4’epi-methylamino-4’-deoxy avermectin B_1a_ and a maximum of 10% 4”-epi-methylamino-4”-deoxy avermectin B_lb_ benzoate [19]. EMB is a very useful biopesticide, is broadly used in the world for controlling insect pests, and is at the same time safe to nontarget organisms [20]. However, the conventional formulations of EMB are highly sensitive to light and ultraviolet (UV) irradiation under natural conditions; therefore, its bioavailability is limited in field applications [21]. Thus, it is necessary to develop a novel formulation of EMB with higher efficacy and prolonged protection. 

In this work, different NFs based on nano-delivery systems for EMB were prepared using CNCs and SNPs as carriers and verified by various techniques such as FE-SEM, TEM, particle size and zeta potential (ZP). Also, the entrapment efficiency (EE), absolute recovery (AR), and UV stability of the EMB + NFs were studied. Furthermore, the toxicity of EMB + NFs was tested against the cotton mealybug, *Phenacoccus solenopsis* Tinsley (Hemiptera: Pseudococcidae), and compared with that of commercial EMB product. The results showed that the NFs improved the photostability of the EMB active ingredient. Additionally, insecticidal activity of EMB + NFs was highly effective against *P. solenopsis* in comparison to the commercial formulation.

## 2. Results and Discussion

### 2.1. Preparation and Characterization of the EMB + NFs

**Figure 1:**
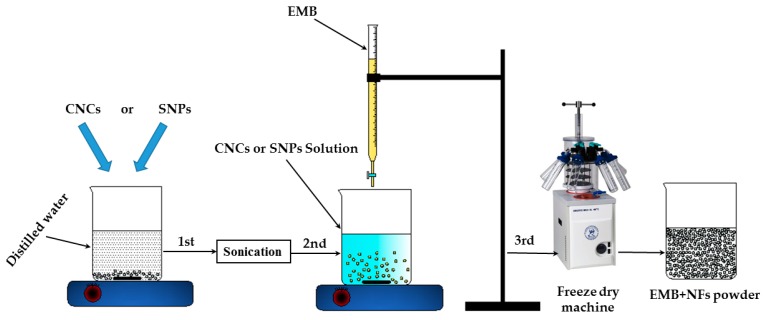
Schematic diagram of the preparation of the emamectin benzoate (EMB) + nanoformulations (NFs).

**Figure 2:**
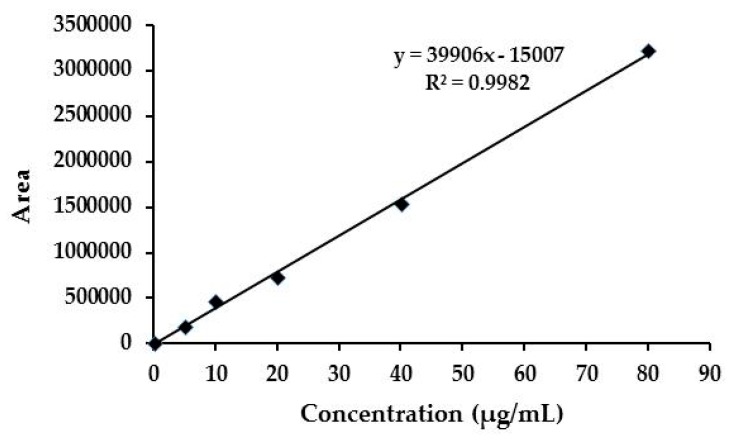
Standard curve of emamectin benzoate.

In this paper, two new EMB nanoformulations, EMB + CNCs and EMB + SNPs, were prepared using CNCs and SNPs as carriers. The preparation process includes three key steps, as illustrated in Figure 1. First, CNCs or SNPs were dispersed in ddH_2_O and sonicated to ensure that CNCs or SNPs wholly dispersed. Then, the EMB solution was added drop by drop into the dispersed CNCs or SNPs under continuously stirred condition by the magnetic stirrer. Finally, the mixture was dried to get EMB + NFs. The standard curve of EMB (Figure 2) displays an excellent linear relationship between the concentrations that range from 5 to 80 μg mL^−1^ with the linear equation y = 39,906x − 15,007 (R^2^ = 0.9982).

The AR was determined by HPLC, comparing the EMB reference values with the total content quantified in the NFs. EMB reference values were calculated by considering the quantity of EMB initially present in the amount of NFs that was used in the processes. The EE was estimated as the ratio between the EMB in NFs and the AR. The % AR of NFs were 69.8% and 93.2%, and the % EE was 87.5% and 94.6% from EMB + CNCs and EMB + SNPs, respectively (Table 1). The high EE of CNCs for EMB (93.2%) was probably due to the large surface area of CNCs. Additionally, Liu et al. [22] stated that CNCs prepared by the acid hydrolysis method have great adsorption ability because of the sulfate groups on the surface of CNCs, and they found that adsorption behavior relies on pH with the finest adsorption obtained around neutral pH. Lin et al. [23] studied the effect of three polysaccharides like starch nanocrystals, chitin whiskers, and CNCs on the drug release properties of sodium alginate microspheres, and they found that the EE for theophylline in the four microspheres was more than 55%. The good EE of SNPs for EMB (87.5%) may also be due their high surface area to volume ratio. As EMB has low solubility in water (24 mg L^−1^) [20], its capacity to be adsorbed onto SNPs’ surfaces is higher than its solubility in water. Besides, silica gel is also used as an adsorbent in the environmental remediation, wastewater treatment, and as a stationary phase in chromatographic columns because silica possesses high adsorption capacity [24]. Our results were in good agreement with Song et al. [17], who found that the amount of chlorfenapyr-loaded on silica nanoparticles was 39.78% and that the chlorfenapyr might be physically adsorbed on the silica nanoparticles. Also, the SNPs illustrated a high loading ability for emamectin benzoate (87.5%) [18]. Consequently, these new nano-carriers such as CNCs and SNPs can be used to improve EMB insecticidal toxicity, solubility, and photodegradation and to overcome the disadvantages of EMB as mentioned earlier.

#### 2.1.1. Morphology Analysis

**Figure 3:**
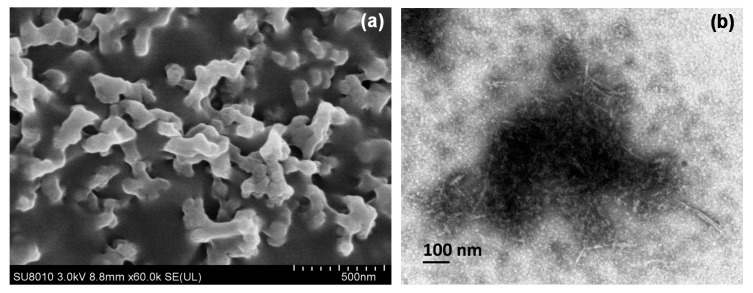
FE-SEM image of CNCs (**a**) and TEM image of EMB + CNCs (**b**).

FE-SEM and TEM pictures of CNCs and EMB + CNCs are presented in Figure 3. The FE-SEM image of CNCs shows some agglomerations and irregular rod-like in form, as shown in Figure 3a. Regarding the EMB + CNCs, the TEM photograph of CNCs shows a uniform structure with a rod-like shape. The mean diameter of CNCs (loaded with EMB) is about 8.9 ± 1.7 nm in width and 78.4 ± 19.1 nm in length (Table 1). The obtained results were compatible with Kargarzadeh et al. [25] who found some agglomerations in the CNCs, which were formed possibly during the water evaporation step and freeze-drying process. In another study, Lin et al. [10] reported that nanocrystals were aggregated because of their hydrophilic nature, strong inter-particle hydrogen bonding, and a large surface area. Similarly, CNCs showed rod-like shapes and had a length of 200–300 nm and width of 10–20 nm [23,26,27].

**Figure 4:**
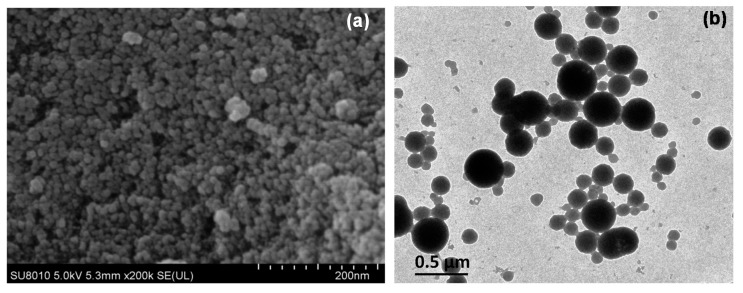
FE-SEM image of SNPs (**a**) and TEM image of EMB + SNPs (**b**).

Figure 4a,b displays an FE-SEM image of the SNPs and a TEM micrograph of the EMB + SNPs, respectively. The FE-SEM image shows that the SNPs are loose aggregates of small particles (Figure 4a). Concerning EMB + SNPs, the TEM photograph exhibits that the SNPs are spherical in shape and the mean particle size is approximately 82.5 ± 9.8 nm; this size is similar to those reported by other scientists [28,29]. TEM image also illustrates that the EMB is adsorbed on SNP surfaces (Figure 4b). 

#### 2.1.2. X-ray Diffraction

**Figure 5:**
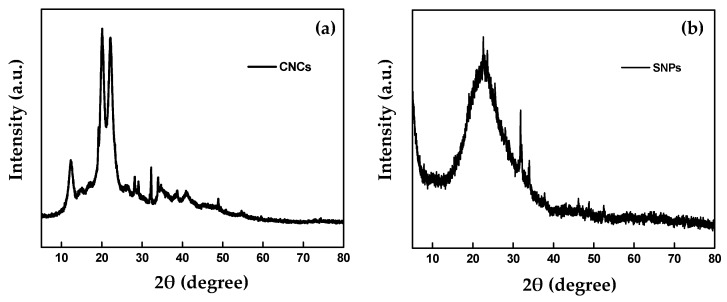
XRD patterns of CNCs (**a**) and SNPs (**b**).

The XRD patterns were used to investigate the crystal structure of CNCs and SNPs. The XRD pattern diagram of CNCs (Figure 5a) clearly shows that the sharp diffraction peaks of the 2*θ* angles at about 12.0°, 20.1°, and 22.1° were attributed to the (110), (210), and (200) planes, respectively. This result was similar to those reported by Naduparambath et al. [30]. The SNPs peaks were observed at 2*θ* = 22.5°, 31.6°, and 34.0° by Bragg’s law, that is, λ = 2d Sin *θ* (Figure 5b). These findings exhibit a single broad peak at 2*θ* = 22.5° for the amorphous SNP core region. This broad peak could be due to the small size and the incomplete internal structure of SNPs. Our results were in accordance with a report by Dubey et al. [31], who observed a broad peak of SNPs that corresponded to the amorphous structure of SNPs. Also, they found that this broad peak of SNPs was probably attributed to the small particle size and a high percentage of amorphous SNPs.

#### 2.1.3. FTIR Analysis

**Figure 6:**
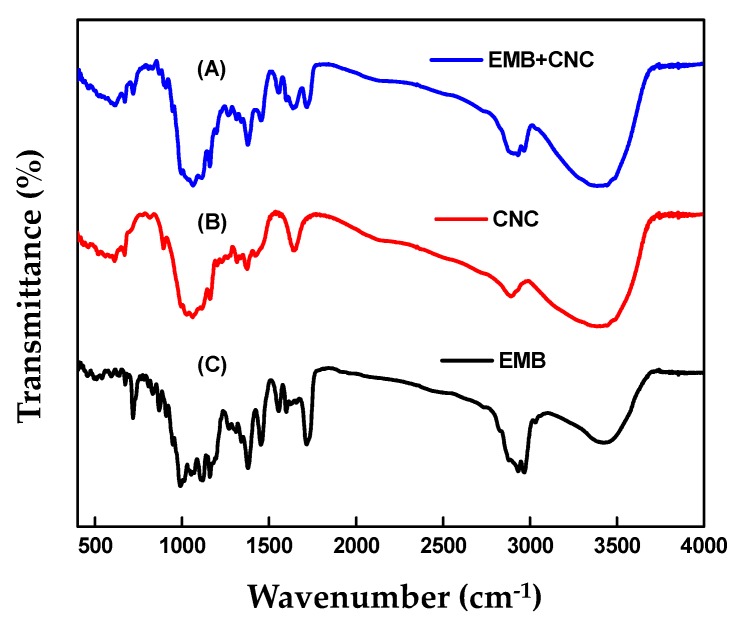
FTIR spectra of EMB + CNCs (**A**), CNCs (**B**) and EMB (**C**).

**Figure 7:**
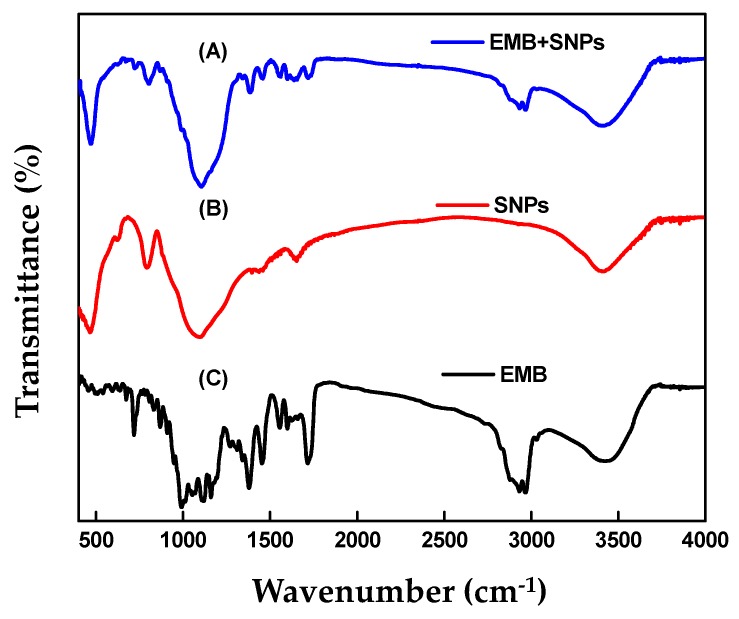
FTIR spectra of EMB + SNPs (**A**), SNPs (**B**), and EMB (**C**).

Figure 6 shows the FTIR spectra of EMB + CNCs, CNCs, and free EMB. Concerning CNCs, the broad peak at 3385 cm^−1^ is assigned to the stretching vibration of the O–H groups (Figure 6B). The peaks at 2890 cm^−1^ correspond to C–H stretching vibration and 1647 cm^−1^ originated from the adsorbed water (H_2_O). The peaks observed at 1421, 1375, and 1316 are because of the bending of CH, CH_2_, and OH respectively, which are typical for polysaccharides, and 1161 cm^−1^ is because of asymmetric vibrations (C–O–C). The peaks at 1060 cm^−1^ and 894 cm^−1^ are ascribed to C–O stretching of the pyranose ring skeleton and the glycosidic linkages between glucose units in cellulose [30,32,33]. Regarding the free EMB (Figure 6C), the peaks at 2967 cm^−1^ and 2932 cm^−1^ are attributed to C–H stretching vibrations of an aromatic ring corresponding to the benzoate fraction or conjugated olefins and the peak at 1716 cm^−1^ is attributed to bending vibration of C=O stretching vibrations of an arylic ester. The bands at 1637, 1599, and 1555 cm^−1^ were assigned to C=C stretching vibrations of an aromatic ring or conjugated olefins, the peak at 1453 and 1379 cm^−1^ are identified as skeleton vibration of C–H deformation in CH_3_ groups. The peaks at 1160, 1118, and 1058 cm^−1^ are ascribed to C–O stretching vibrations, to O–H and C–O–C flexion, the peak at 990 cm^−1^ is attributed to bending vibration of C–H flexion of trans C=C bonding, and the peak at 947–510 cm^−1^ is assigned to C–H flexion outside the plane in an aromatic ring or C=C cis bond. In the case of EMB + CNCs (Figure 6A), the spectrum reserved most of the main peaks of pure CNCs and EMB and no remarkable new peaks were observed. Figure 7 displays FTIR spectra of EMB + SNPs, SNPs, and free EMB. For SNPs, the peak at 3416 cm^−1^ shows that a small quantity of water is present in samples (Figure 7B). The emphatically broad peak at 1094 cm^−1^ is assigned to the composite of Si–O stretching of SNPs. The FTIR spectrum of free EMB is mentioned above (Figure 7C), and in the case of EMB + SNPs (Figure 7A), no notable new peaks were observed. These results confirmed the EMB + NFs spectra reserved most of the main peaks of pure CNCs, SNPs, and free EMB, and no remarkable new peaks are observed, suggesting that most of the adsorption of EMB on the CNCs and SNPs carriers is probably physical and that the EMB properties were unchanged after loading. The results were consistent with the adsorption behavior of CA/AVM (avermectin-cellulose acetate) reported by Zhao et al. [34].

#### 2.1.4. Thermogravimetric Analysis

**Figure 8:**
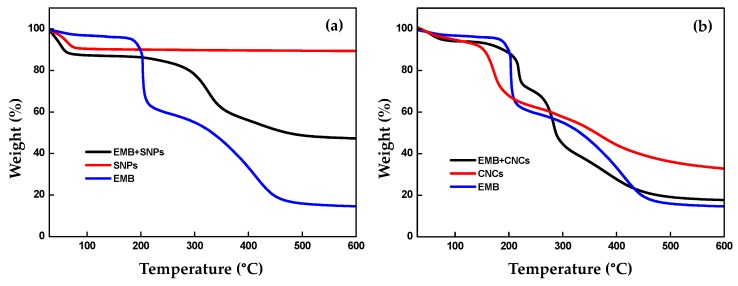
TGA curves for SNPs, EMB + SNPs, EMB (**a**), CNCs, EMB + CNCs and EMB (**b**).

The amount of EMB loaded on CNCs and SNPs was measured by thermogravimetric analysis (TGA). Figure 8a,b shows the TGA curves of SNPs, EMB + SNPs, CNCs, EMB + CNCs, and EMB technical. The results demonstrate that the weight loss before 150 and 111 °C is possibly ascribed to the evaporation of water in the samples. While the weight loss between 150 °C to 247 °C and 115–241 °C is mainly attributed to volatilization and decomposition of EMB. The total weight losses for SNPs, EMB + SNPs, CNCs, and EMB + CNCs in the range of 150–600 °C and 115–600 °C were about 9.69%, 53.00%, 67.46%, and 82.50%, respectively. Therefore, the loading efficiency of EMB was approximately 43.31% and 15.04% for EMB + SNPs and EMB + CNCs, respectively, indicating that EMB was loaded onto SNPs and CNCs nanocarriers.

#### 2.1.5. Brunauer–Emmett–Teller (BET) Analysis

**Figure 9:**
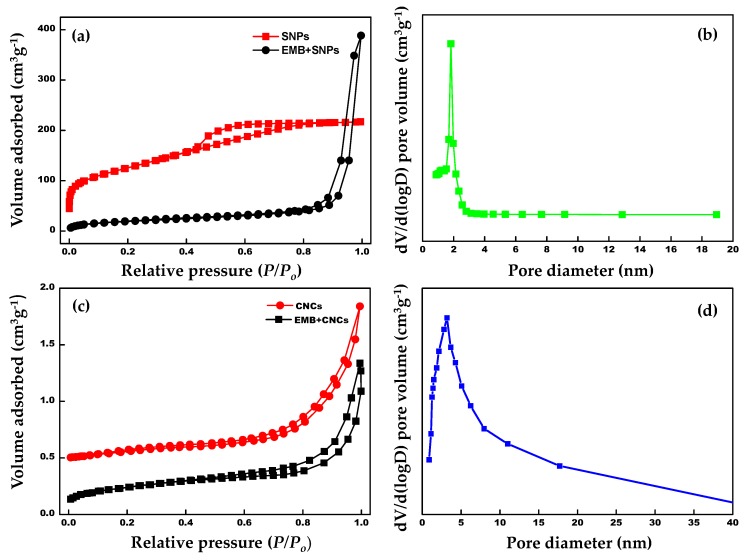
(**a**,**c**) Nitrogen adsorption–desorption isotherms of SNPs, EMB + SNPs, CNCs, and EMB + CNCs and (**b**,**d**) pore diameter distribution of SNPs and CNCs.

The surface area and corresponding pore size distribution of the samples were investigated by nitrogen adsorption–desorption measurements (Figure 9a–d). As can be seen from Figure 9a,c, the samples reveal a typical IV type with H_3_ hysteresis loop. The surface areas analyzed based on the Brunauer–Emmett–Teller (BET) method and pore volumes are of the samples are summarized in Table 2. Moreover, the formation of the mesoporous structure of the template samples (SNPs and CNCs) can be validated by the wider loops within the high relative pressure zone. The pore size distribution spectra optimized by the Barrett–Joyner–Halenda (BJH) theory (Figure 9b,d) indicate preferable mesoporous features. The BET surface area and pore diameter for pristine SNPs were found to be 237.5 m^2^ g^−1^ and ~1.8 nm, respectively. After loading EMB onto SNPs, the BET surface area of EMB + SNPs and pore volume was decreased to 69.4 m^2^ g^−1^ and 0.157 cm^3^ g^−1^, respectively. In the case of blank CNCs, the specific surface area and pore size were approximately 13.2 m^2^ g^−1^ and 3.3 nm, respectively. The specific surface area of EMB + CNCs was reduced to 3.9 m^2^ g^−1^, which indicates the successful loading of EMB onto hierarchal CNCs. It is noteworthy that the adsorption capacities and specific surface areas of the naked NPs decreased significantly after EMB loading onto SNPs and CNCs. These results support the fact that pores of SNPs and CNCs were fully occupied by EMB molecules.

#### 2.1.6. Zeta Potential

Zeta potential is an important technique for measuring the surface charge and physical stability of nanoparticles in the aqueous medium. Nanoparticles with zeta potential values of higher than +30 mV or less than −30 mV are considered to be highly stable in the colloidal medium [35]. In the present work, the zeta potential values of the EMB + CNCs and EMB + SNPs are found to be about −26.4 ± 2.9 and −30.0± 0.7 mV, respectively (Table 1). The negative surface charge of CNCs was assigned to the existence of sulfate groups after sulfuric acid hydrolysis [26,30]. According to obtained results, zeta potential displayed a negative charge on the surface of nanoformulations, which is an acceptable range for showing physical stability. Hence, the two nanoformulations prepared in this study are considered to be highly stable in the aqueous medium.

### 2.2. Stability Study

**Figure 10:**
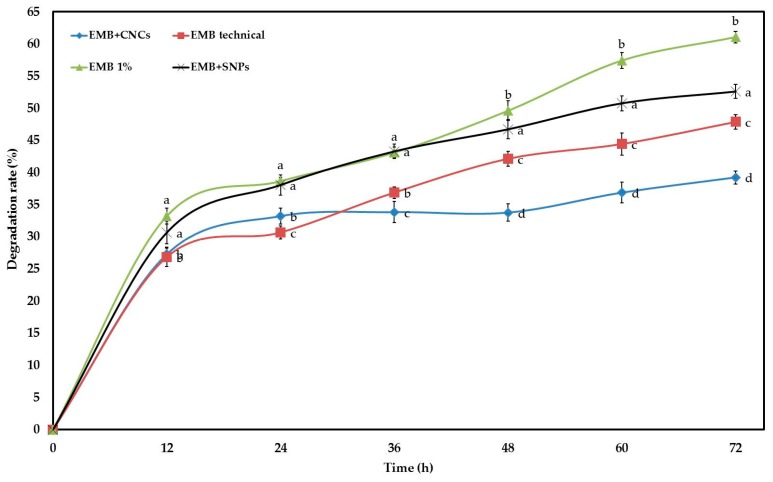
Photodegradation curves of EMB + CNCs, EMB (technical), EMB 1%, and EMB + SNPs under UV irradiation. Means at the same time followed by the same letters are not significantly different at *p* = 0.05 (LSD test). Mean is calculated from three repetitions.

EMB is degraded rapidly in the field due to UV radiation. Therefore, CNCs and SNPs were used as carriers to improve EMB stability against photolysis and extend half-life time. The degradation rates were 39.23, 47.87, 52.60, and 61.07% for EMB + CNCs, EMB technical, EMB + SNPs, and commercial EMB EC after 72 h, respectively (Figure 10), with these values differing significantly with each other. The obtained results suggest that EMB + CNCs were more stable than other samples, with the exception that their stability is similar to EMB technical at 12 h and lower than EMB technical at 24 h after exposure to ultraviolet irradiation. The stability of EMB + SNPs was always lower than that of EMB technical, and it is better than that of EMB 1% only after exposure to ultraviolet irradiation for 48 h or more hours. These results clearly show that CNCs can protect EMB well and can improve the photostability of EMB. The stability of EMB + CNCs against UV irradiation could be due to the chemical and physical properties of CNCs, such as high surface to volume ratios, good mechanical properties, low thermal expansion, and active functional groups on the surface, which can readily be chemically modified to give additional functionalities and to enhance dispersion in solvents and biocompatibility. Also, the TEM image shows that the CNCs contain networked structures, which can protect EMB from photolysis. These findings were consistence with the results of Nguyen et al. [36], who found that chitosan-coated beeswax solid lipid nanoparticles (CH-BSLNs) displayed good protection for deltamethrin against photolysis. Moreover, CNCs are widely used in biomedical applications, gene vectors, and a drug delivery system since it has remarkable physical properties and excellent biological properties (low toxicity, biodegradability, and biocompatibility) [37,38]. Probably, the reason why SNPs cannot protect EMB technical from UV is that the EMB technical was adsorbed on the external surface of SNPs. Consequently, we can use this nanoformulation (EMB + CNCs) under adverse climatic conditions to protect crops for a long time and to maybe also decrease economic cost by reducing pesticide dosage and the number of application times.

### 2.3. Bioactivity

The biological activities of NFs and commercial EMB EC against *P. solenopsis* are shown in Table 3. The LC_50_ value of the EMB + SNPs was 0.01 μg mL^−1^ after 48 h, whereas the LC_50_ for EMB + CNCs and commercial EMB EC were 0.05 and 0.31 μg mL^−1^ after 72 h, respectively, by the leaf dip method. Toxicity of the EMB + SNPs and EMB + CNCs against *P. solenopsis* were 63.0 and 9 folds of the EMB EC formulation, respectively, after 48 h, and the toxicity of EMB + CNCs was 6.2-fold of EMB EC formulation at 72 h after exposure. These results demonstrated that the NFs were more efficient than commercial EMB EC formulation, suggesting that SNPs and CNCs as carriers can considerably improve the insecticidal activity of EMB. The reason why NFs are much better than traditional pesticide formulations against *P. solenopsis* is probably that their high surface area and the small particle size might increase the penetration and absorption of the active ingredient in the insect body. Furthermore, the surface-functionalized silica nanoparticles can deliver DNA and drugs into animal cells and tissues [39] because nanoparticles drug carriers have the potential to cross physiological barriers and access different tissues [40]. These results concur with a report by Meyer et al. [41], who found that the Functional Nano-Dispensers (FNDs) of imidacloprid was about a 200-fold reduction in the quantity of imidacloprid active ingredient needed to achieve similar mortality of *Diaphorina citri* as compared with the commercial formulation. Similar results were also reported by Saini et al. [42]; they mentioned that the biological activity of pyridalyl nanocapsules was more efficient than the commercial product on *H. armigera* larvae and that increased toxicity of nanosized formulation on larvae maybe is because of increasing penetration of pyridalyl in the larval body. In another work, Kaziem et al. [43] reported that the biological activity of AVM-CRF was 40% higher than AVM commercial formulation against *P*. *xylostella*. Emamectin benzoate-conjugated polyacrylate nanoparticles (EB-CN SE) also showed much better control effects than the commercial product (emamectin benzoate EC) on *H. armigera* [44].

## 3. Materials and Methods

### 3.1. Materials

The model insecticide, technical grade EMB (70%) was provided by Shanghai Bosman Industrial Co., Ltd., (Shanghai, China). EMB 1% emulsifiable concentrate (EC) was obtained from Syngenta Crop Protection Co., Ltd. (Suzhou, China). Cellulose, sodium silicate (Na_2_SiO_3_), sulfuric acid (H_2_SO_4_), and acetic acid were obtained from Sangon Biotech Co., Ltd., (Shanghai, China). Dichloromethane, methanol, and acetonitrile were of high-performance liquid chromatography (HPLC) grade and purchased from Sigma-Aldrich (St. Louis, MO, USA). Milli-Q water (18.2 MΩ cm, TOC ≤ 4 ppb) was employed in all analytical experiments.

#### Insect Culture

*Phenacoccus solenopsis* was collected from ornamental plants in the eastern suburbs of Hangzhou, China, in June 2017. The cotton mealybug population was reared on tomato plants in cages and was maintained at 27 ± 2 °C and 65 ± 5% R.H. with a 14:10 (L:D) photoperiod in the laboratory of Insect Ecology and IPM, Institute of Insect Sciences, Zhejiang University.

### 3.2. Preparation of EMB + NFs

#### 3.2.1. Synthesis of CNCs and Loading EMB

CNCs were prepared using the method of Beck-Candanedo et al. [45]. Ten grams of cellulose was added into a 100-mL 64% sulfuric acid (H_2_SO_4_) solution (*w*:*w*) and hydrolyzed at 45 °C for one hour under vigorous magnetic stirring. The acid hydrolysis was stopped by diluting the reaction tenfold with chilled double distilled water (ddH_2_O). The hydrolysis solution was centrifuged at 11,000 rpm for 15 min. The sediments were washed with ddH_2_O four times to reduce acid concentration and then resuspended in ddH_2_O and dialyzed until pH reached 7.0. During the dialysis, the sample was sonicated for 10 min in an ice bath to overcome overheating and then freeze-dried by using a machine (Alpha 1–2 LD plus; Martin Christ Gefriertrocknungsanlagen GmbH, Osterode am Harz, Germany) to get CNC powder.

The EMB + CNCs were synthesized using a freeze-drying method [46]. Firstly, 1.0 g of CNCs was dispersed in 250 mL of ddH_2_O and the CNCs were sonicated for 30 min to ensure that CNCs have entirely dispersed. Then, 1.42 g of EMB technical grade (equivalent to 1 g EMB active ingredient) was dissolved in 20 mL of methanol. The EMB solution was added dropwise into the dispersed CNCs and continuously stirred by the magnetic stirrer at a speed of 800 rpm for two hours at 25 °C. After the EMB was lodged entirely onto the surface of CNCs, the mixture was freeze-dried for 24 h to obtain EMB + CNCs powder.

#### 3.2.2. Synthesis of SNPs and Loading EMB

The SNPs was prepared by a sol-gel method with some modifications, as described by Musić et al. [47]. Approximately 0.2 g equivalent sodium silicate (Na_2_SiO_3_) was diluted in 300 mL of ddH_2_O and 0.2 g equivalent H_2_SO_4_ was diluted in 200 mL of ddH_2_O. Then, the H_2_SO_4_ solution was added drop by drop into the sodium silicate solution. The mixture was stirred continuously by the magnetic stirrer for 45 min to get SNP gel. The SNP gel was washed five times with ddH_2_O in a filter paper to remove impurities from the mixture under vacuum filtration. The SNP gel was dried by using a machine Alpha 1–2 LD plus for 24 h. Finally, the dried sediment was calcined at 600 °C for two hours to get SNP powder.

The EMB + SNPs were prepared using a freeze-drying method. Firstly, 1.0 g of SNPs was dispersed in 250 mL of dd H_2_O and the SNPs were sonicated for 30 min to ensure that SNPs have entirely dispersed. Then, 1.42 g of EMB technical grade (equivalent to 1 g EMB active ingredient) was dissolved in 20 mL of methanol. The EMB solution was added dropwise into the dispersed SNPs and continuously stirred by the magnetic stirrer at a speed of 600 rpm for two hours at room temperature. The mixture was dried by the same method mentioned above to obtain EMB + SNPs powder.

### 3.3. Characterization

The morphologies of CNCs and SNPs were studied using field emission scanning electron microscopy (FE-SEM, TM-1000, Hitachi, Japan) with the accelerating voltage of 3.0 and 5.0 kV. A thin layer of the samples was prepared on the carbon coated copper grid by merely dropping a small quantity of the samples on the grid. The samples were coated with gold by a vacuum sputter coater before the examination to avoid charging during FE-SEM observation. Transmission electron microscopy (JEM-1230, JEOL, Akishima, Japan) was used to study the structure and to measure the particle sizes of CNCs, SNPs, and EMB + NFs. The samples were prepared via dispersion in distilled water, and a drop of the diluted solution was placed onto a carbon-coated copper grid and then allowed to dry at room temperature. X-ray diffraction (XRD) patterns of the powder were performed using an X’PERT-PRO-PANalytical apparatus (PANalytical, Almelo, The Netherlands) with Cu-K*α* radiation (λ = 0.15406 nm) to characterize the structure of the prepared nanoparticles. The diffraction results were recorded in the range of 10–80° at the 2*θ* angle with a resolution of 0.02°. A Fourier transform infrared spectrophotometer (FTIR) (Vector 22, Bruker, Ettlingen, Germany) was used to characterize the various functional groups in the samples. The FTIR spectra of CNCs, SNPs, and EMB were recorded in the range of 400–4000 cm^−1^ region at a resolution of 4 cm^−1^. Thermogravimetric analysis (TGA) was conducted to determine the loading efficiency of EMB by using an SDT Q600 (TA Instruments-Waters LLC, New Castle, DE, USA) apparatus from 25 to 600 °C with a heating rate of 10 °C/min under nitrogen atmosphere. The Brunauer–Emmett–Teller (BET) surface area and pore size distribution of the as-synthesized CNCs, EMB + CNCs, SNPs, and EMB + SNPs were determined by nitrogen adsorption-desorption isotherms, which were performed at −196 °C on a Micromeritics ASAP 2460 analyzer (Micromeritics Instrument Corporation, Norcross, GA, USA). The BET model and Barrett–Joyner–Halenda (BJH) method were used to estimate the specific surface area and pore size distribution of the samples, respectively. A Zetasizer Nano ZS90 (Malvern Instruments Ltd., Malvern, UK) was used to measure the zeta potential by a dynamic light scattering (DLS) at 25 °C. Each sample was measured three times.

### 3.4. Quantitative Determination of Emamectin Benzoate in NFs

The following technique was used to determine the total amount percentages of absolute recovery (AR) of EMB in the two nanoformulations. First, 1 mL of the EMB + CNCs or EMB + SNPs mixture was dissolved in 10 mL of dichloromethane and the samples were sonicated for 30 min. The mixture was magnetically stirred at 1000 rpm for three hours to ensure complete extraction of the EMB from samples. After that, 1 mL of the samples were centrifuged (Centrifuge 5417 R; Eppendorf, Hamburg, Germany) at 20,000 rpm for 30 min at room temperature. After phase separation, 0.1 mL of the supernatant was dried under vacuum (Concentrator plus, Eppendorf, Hamburg, Germany). The dried compound containing EMB was redissolved in 1 mL of methanol, and the total quantity of EMB was determined by HPLC (Agilent HP1260, Santa Clara, CA, USA) with UV detection at 245 nm. The chromatographic conditions were as follows: C18 column (250 × 4.6 mm, id, 5 μm) was used with a mobile phase containing acetonitrile and water with 0.1% acetic acid (80:20, *v*/*v*). The injection volume was 20 μL, and the flow rate was 1.0 mL/min.

The percentages of entrapment efficiency (%EE) of EMB in the two nanoformulations were estimated by measuring the concentration of the free unloaded compound in the aqueous medium of the EMB + CNCs and EMB + SNPs. Centrifugation was performed by a tube filter containing a 0.22-µm pore cellulose acetate membrane (Costar Spin-X, Corning Inc., Louis, MO, USA). About 0.5 mL of EMB + CNCs or EMB + SNPs suspension was placed in the outer chamber of the filter assembly, and the assembly was then centrifuged at 2800 rpm for 15 min at 15 °C. The nanoparticles were retained in the external room, while the aqueous phase containing the free unloaded EMB was moved to the sample recovery chamber via the filter membrane. After the separation step, 0.2 mL of the aqueous medium was dried. The dried compound was redissolved in 1 mL of methanol, and the concentration was determined by HPLC, according to Forim et al. [1]. Then, the %EE was calculated by using the following equation:

(1) $$EE\;\left(\%\right)=\frac{\left(\mathrm{total}\;\mathrm{quantity}\;\mathrm{of}\;\mathrm{EMB}\;-\;\mathrm{the}\;\mathrm{quantity}\;\mathrm{of}\;\mathrm{free}\;\mathrm{EMB}\;\mathrm{in}\;\mathrm{the}\;\mathrm{aqueous}\;\mathrm{medium}\right)}{\left(\mathrm{total}\;\mathrm{quantity}\;\mathrm{of}\;\mathrm{EMB}\right)}\;\times 100$$

### 3.5. Stability Test

The stability of the NFs against ultraviolet irradiation was evaluated as follows: 0.2 g of EMB technical grade and commercial EMB were dissolved in 250 mL of methanol-water mixture (30:70, v/v), and the EMB + NFs containing the same amount of EMB was diluted with a methanol–water mixture (30:70, *v*/*v*) to the same concentration of EMB. The specimens were exposed to a 36 W germicidal lamp (254 nm) at a distance of 20 cm at 25 °C. After that, 2 mL of the samples were withdrawn every 12 h within the 72 h, followed by centrifugation and using the supernatant to perform HPLC analysis to estimate the active ingredient changes in the NFs compared with the EMB technical grade and commercial EMB that were used as a control in the test [48].

### 3.6. Bioassay

Leaf dipping method was employed to assess the biological activity of two NFs against 2nd instar nymphs of *P*. *solenopsis* [49]. Different concentrations of EMB (two nanoformulations and commercial formulation) were prepared based on the mortality range falling between 20 and 80% [50]. Tomato leaves were immersed into the NFs or commercial EMB 1% EC solution for 20 s and allowed to dry at room temperature for 2 h. In every petri dish, a dried leaf was put into the petri dish (5 cm in diameter) with a piece of moist filter paper used to prevent the dryness of the leaves. Ten 2nd instar nymphs of *P. solenopsis* were introduced into each petri dish, and each treatment was repeated three times. The leaves for control treatment were dipped into water only. All bioassay was carried out under the same laboratory conditions as mentioned above. Insect mortality was evaluated 24, 48, and 72 h after exposure to various concentrations of NFs and commercial EMB 1% EC. Cotton mealybug nymphs were considered dead if they failed to display any leg movement when gently touched by camel hairbrush.

### 3.7. Statistical Analysis

The degradation data were analyzed using one-way analysis of variance (ANOVA) with SAS Statistics 9.1 software [51], and the means were compared by the least significant difference (LSD) test. The degradation percentage data were transferred via arcsine square root before ANOVA to standardize means and normalize variances and were transformed back to percentages for presentation. Results with *p* (probability) less than 0.05 were deemed to be statistically significant. The bioassay data were analyzed with the SPSS software package (version 22.0) by probit analysis [52] to calculate LC_50_ values, 95% confidence limits (CLs), slope, and Chi-square (χ^2^).

## 4. Conclusions

In summary, we successfully prepared different NFs (EMB + CNCs and EMB + SNPs) based on nano-delivery systems by loading EMB with CNCs and SNPs as carriers to improve chemical stability and biological activity of EMB. FE-SEM, TEM, XRD, TGA, BET, and FTIR spectroscopy confirmed the formation of the EMB + NFs. The results displayed that EMB + NFs had a high loading efficiency for EMB (approximately 43.31% and 15.04% w/w for SNPs and CNCs, respectively). EMB + NFs can effectively protect the EMB active ingredient from photodegradation. Moreover, the bioassay results showed that NFs were highly effective against *P. solenopsis.* In agricultural applications, pesticide usage could be decreased using the EMB + NFs by reducing the recommended dose of pesticides and the number of application times, thereby reducing the residue of pesticides in food as well as minimizing the hazard of environmental pollution and the harms to farmers.

## Figures and Tables

**Table 1:** Characterization of cellulose and silica nanoparticles loaded with EMB.

Formulation	Zeta Potential (mV)	Particle Size		Entrapment Efficiency (%)	Absolute Recovery (%)
		Width (nm)	Length (nm)		
EMB + CNCs ^a^	−26.4 ± 2.9	8.9 ± 1.7	78.4 ± 19.1	93.2	69.8
EMB + SNPs ^b^	−30.0 ± 0.7	82.5 ± 9.8	-	87.5	94.6

^a^ CNCs: Cellulose nanocrystals, ^b^ SNPs: Silicon dioxide nanoparticles.

**Table 2:** Nitrogen adsorption analysis of SNPs, EMB + SNPs, CNCs, and EMB + CNCs.

Sample	S_BET_ (m^2^ g^−1^)	BJH Pore Diameter (nm)	Pore Volume (cm^3^ g^−1^)
SNPs	237.5	1.8	0.196
EMB + SNPs	69.4	-	0.157
CNCs	13.2	3.3	0.0008
EMB + CNCs	3.9	-	0.0001

**Table 3:** Bioassay results of EMB + NFs against the second instar nymphs of *P. solenopsis*.

Formulation	Time (h)	LC_50_ (95% CL, µg/mL)	Slope ± SE	χ^2^
EMB + SNPs	24	0.05 (0.03–0.07)	1.52 ± 0.37	1.27
	48	0.01 (0.003–0.02)	1.70 ± 0.48	0.81
EMB + CNCs	24	0.13 (0.10–0.19)	1.92 ± 0.38	1.99
	48	0.07 (0.05–0.10)	1.91 ± 0.39	2.59
	72	0.05 (0.03–0.07)	2.09 ± 0.42	3.51
EMB 1% EC	24	2.91 (1.57–7.95)	0.81 ± 0.25	0.12
	48	0.63 (0.11–1.19)	0.86 ± 0.26	0.16
	72	0.31 (0.02–0.66)	0.98 ± 0.29	0.69
